# Antimicrobial Susceptibility of Enterococci Isolated from Nestlings of Wild Birds Feeding in Supplementary Feeding Stations: The Case of the Canarian Egyptian Vulture

**DOI:** 10.3390/pathogens13100855

**Published:** 2024-10-01

**Authors:** Margarita Rosa González-Martín, Alejandro Suárez-Pérez, Alejandro Álamo-Peña, Carmen Valverde Tercedor, Juan Alberto Corbera, María Teresa Tejedor-Junco

**Affiliations:** 1 Research Institute of Biomedical and Health Sciences, University of Las Palmas de Gran Canaria, Paseo de Blas Cabrera Felipe “Físico” s/n, 35016 Las Palmas de Gran Canaria, Canary Islands, Spain; margaritarosa.gonzalez@ulpgc.es (M.R.G.-M.); alejandro.suarezperez@ulpgc.es (A.S.-P.); alealape27@gmail.com (A.Á.-P.); carmenvalter@gmail.com (C.V.T.); mariateresa.tejedor@ulpgc.es (M.T.T.-J.); 2 Department of Clinical Sciences, University of Las Palmas de Gran Canaria, 35001 Las Palmas de Gran Canaria, Canary Islands, Spain; 3 Gestión y Planeamiento Territorial y Medioambiental, S.A. (GESPLAN), Gobierno de Canarias, 35003 Las Palmas de Gran Canaria, Canary Islands, Spain; 4 Hospital Clínico Veterinario, University of Las Palmas de Gran Canaria, 35413 Arucas, Gran Canaria, Canary Islands, Spain

**Keywords:** antimicrobial resistance, Enterococci, Canarian Egyptian vulture, multidrug resistance, One Health, wildlife surveillance

## Abstract

Antimicrobial resistance is a growing concern worldwide, requiring a holistic “One Health” strategy to address the interconnectedness of human, animal, and environmental health. This study focused on Enterococci isolated from Canary Island Egyptian vulture chicks, an endangered species that feeds at supplementary feeding stations in the Canary Islands. Sampling and identification revealed the presence of several *Enterococcus* species, with a predominance of *E. faecalis*. Antimicrobial susceptibility testing showed resistance patterns, especially to important antibiotics such as quinolones, vancomycin, and linezolid. The prevalence of multidrug-resistant profiles was lower than that in other wild bird species. This study underscores the need for further research to understand the dynamics of antimicrobial resistance in wildlife and its implications for public health and conservation efforts, emphasizing the importance of a “One Health” approach to address this pressing problem.

## 1. Introduction

Antimicrobial resistance is a global problem, which must be combated with a comprehensive “One Health” approach, i.e., taking into account that human health should not be dissociated from animal and environmental health [1,2,3]. The increasing prevalence of multidrug-resistant (MDR) or extensively drug-resistant (XDR) bacteria [4] reinforces the need to develop surveillance studies to detect and monitor these strains. Due to their resistance to most antimicrobial molecules, XDR strains have become a cause for concern at all levels in our society.

Wildlife has been identified as one of the drivers of the dissemination of genes conferring resistance to clinically relevant antimicrobials [5,6,7,8]. Although theoretically, wild animals are not exposed to clinically relevant antibiotics, the detection of MDR strains in such wildlife is increasing considerably, reinforcing the importance and need for focused studies on this topic [9,10,11]. In addition, monitoring of these bacteria in wild animals has become an important surveillance tool since it could reflect antimicrobial resistance in strains isolated from humans [12]. However, some authors disagree with this claim by finding inconsistencies in the geographical distribution of resistance patterns in humans and wildlife [13].

Antibiotic resistance may also be a good indicator of human influence on wildlife exposure to MDR bacteria. Animals in the wild can acquire these bacteria from purified water, farms, landfills, etc. [14,15,16,17,18,19], with wild birds being one of the most studied groups in relation to this topic [15,17,20,21,22].

The Canarian Egyptian vulture (*Neophron percnopterus majorensis*) is a subspecies of the Egyptian vulture. It is endemic to the Canary Islands, and it is included in the Spanish Catalogue of Endangered Species under the category “In Danger of Extinction” and under the category “Endangered” in the International Union for Conservation of Nature’s (IUCN) Red List of Threatened Species. It is necrophagous and feeds on the carcasses of dead animals and also obtains food from midden and landfills. Currently, Canary vultures are only found in two of the islands of the Canary Islands Archipelago (Fuerteventura and Lanzarote), where supplementary feeding stations have been installed to assure feed supply (mostly pig carcasses) and contribute to the recovery of this species [23].

Supplementary feeding stations (SFSs) increase the probability of the survival of these birds by ensuring feed supply, but they also represent a risk in terms of exposure to resistant bacteria and drugs [24]. Several authors have demonstrated, for example, the transmission of antibiotic-resistant *Salmonella* to vultures from feeding pig carcasses at supplementary feeding stations [25]. The presence of antibiotics in livestock carrion that may be ingested by necrophagous birds may contribute to the selection of antibiotic-resistant bacteria in their digestive tracts [26,27,28].

Enterococci are ubiquitous microorganisms, and they are usually found as part of human and animal microbiota. However, some species are considered relevant nosocomial pathogens; for instance, *Enterococcus faecium* is included among “ESKAPE” pathogens due to its ability to become MDR [29,30]. Together with *Enterococcus faecalis,* both have great clinical relevance and represent a public health concern [31,32,33]. They are also considered emergent pathogens in animals, especially in poultry [34].

Enterococci present intrinsic or natural resistance to multiple antibiotics and have the ability to quickly develop resistance mechanisms as soon as a new antimicrobial starts to be used, as has been observed for linezolid or quinupristin/dalfopristin [35,36].

Thus, the aim of this study was to assess the diversity of *Enterococcus* species in samples from wild Canarian Egyptian vulture nestlings and to analyze their antimicrobial susceptibility patterns from a “One Health” perspective.

## 2. Materials and Methods

All the Canarian Egyptian vulture nestlings born were sampled (N = 44) in June and July 2023 during ringing activities within a long-term monitoring program of Canarian Egyptian vultures (*Neophron percnopterus majorensis*). Chicks were captured during the fledgling stage from nests. The sampling procedure was carried out following the recommended guidelines for the care and ethical use of animals. All procedures were carried out under the project license approved by the Biodiversity Directorate of the Government of the Canary Islands; the official reference number of the committing authority is 44/2023 (1 February 2023) as an extension of resolution 57/2020.

Nasal (choanal) and cloacal swabs were obtained using a sterile Amies blue plastic/viscose gel transport medium (Darmstadt, Germany) and stored at 4 °C until arrival at the Microbiology Laboratory within 24 h.

To recover Enterococci, cloacal samples were cultured on mEnterococcus (mE) Agar (Difco, St. Louis, MO, USA) and mEnterococcus Agar + Vancomycin 4 µg/mL (mE + VAN). For nasal samples, a previous step of inoculation in Brain–Heart Infusion Broth (BHI, Difco) was carried out, and after overnight inoculation at 37 °C, the samples were seeded on previously indicated media. Plates were incubated for 24–48 h at 37 °C.

On the basis of morphology and color, suspected *Enterococcus* colonies were isolated. At least one of each different suspected *Enterococcus* colony was isolated in new mE or mE + VAN plates. After incubation overnight, Gram stain and catalase tests were conducted. Gram-positive catalase-negative cocci were considered presumptive *Enterococcus* and were later identified by API 20 STREP (bioMérieux, Marcy L’Etoile, France).

Antimicrobial susceptibility was determined by the disk diffusion method using BD BBL disks (BD BBL, Sparks, MD, USA) and following the instructions and recommendations of the Clinical and Laboratory Standards Institute (CLSI) [37]. The antimicrobial agents tested were (in µg/disk) ampicillin (AM 10), ciprofloxacin (CIP 5), levofloxacin (LVX 5), doxycycline (D 30), vancomycin (VAN 30), teicoplanin (TEI 30), linezolid (LZD 30), and quinupristin/dalfopristin (SYN 15). Imipenem (IMP 10) was also tested, and inhibition zones were interpreted following the instructions and recommendations of the European Committee on Antimicrobial Susceptibility Testing (EUCAST) (https://www.eucast.org/eucastguidancedocuments, accessed on 25 July 2024) published by the European Society of Clinical Microbiology and Infectious Diseases. Determination of high-level resistance to gentamicin (HLGR) (GM 500 µg) and streptomycin (HLSR) (S 2000 µg) was performed using the plate dilution technique [37]. *Enterococcus faecalis* ATCC 29212 was used as a reference strain.

All isolates that were non-susceptible to vancomycin by the disk diffusion method were also tested using E-test^®^. Minimal inhibitory concentration (MIC) breakpoints were defined following EUCAST criteria. An isolate was considered to be resistant when its MIC was greater than 4 µg/mL and it was not *E. gallinarum* or *E. casseliflavus.*

An isolate was considered MDR when it presented non-susceptibility to at least one agent in three or more antimicrobial categories from the ones established to be tested for this genus or family. In this context, “non-susceptibility” means that the result obtained from in vitro susceptibility tests is either resistant, intermediate, or non-susceptible. In addition to this, if intrinsic resistance to an antimicrobial is present in a species, this agent or category must be removed from the list before applying the criteria to consider the isolate MDR [4] (Table 1).

## 3. Results and Discussion

We studied *Enterococcus* species’ distribution and the antimicrobial susceptibility of isolates obtained from 88 samples (44 from the choana and 44 from the cloaca) of Canarian Egyptian vulture chicks in nests. One hundred and four presumptive *Enterococcus* species were isolated. Results from API 20 Strep identification yielded two *Streptococcus uberis* isolates (from the cloaca and choana of the same animal) and one *Lactococcus lactis* isolate (from the cloaca). Using API 20 Strep, 72 *E. faecalis* isolates (30 from the choana and 42 from the cloaca), 16 *E. faecium* isolates (14 from the choana and 2 from the cloaca), 8 *E. gallinarum* isolates (4 from the choana and 4 from the cloaca), and 1 *E. casseliflavus* (from the cloaca) isolate were identified. In addition to this, four isolates that could not be identified were sent to the University of La Rioja to be tested by MALDI-TOF. One of each of the following species was identified: 1 *E. faecalis* (from the cloaca), 1 *E*. *faecium* (from the choana), 1 *E. gallinarum* (from the cloaca), and 1 *E. casseliflavus* (from the choana).

*E. faecalis* accounted for 72.3% of the species isolated, followed by *E. faecium* (16.8%), *E. gallinarum* (8.9%), and *E. casseliflavus* (2%). All species except *E*. *faecium* were present in similar proportions in cloacal and choanal samples. In a study on fecal samples from free-living and captive raptors [38], 95% of Enterococci isolates were identified as *E. faecalis*; no *E. faecium* was found, and only one isolate was identified as *E. gallinarum*. Abdullahi et al. [14] found that 69.2% of stork nestlings’ nasal samples were positive for Enterococci, with *E*. *faecalis* being one of the most frequent species of bacteria isolated in this kind of sample (48.5%). They also found that *E. faecium* had a higher probability of being found in nasal samples from nestlings of parent storks foraging in landfills. Other authors [39,40] also found *E. faecalis* to be the most frequent species in fecal and pellet samples from wild birds. Species distributions were clearly different when raptors from a recovery center were tested [41], revealing that *E. faecium* accounted for 64.71% and *E. faecalis* for 29.41% of isolates from dead raptors’ fecal samples. In fecal samples obtained from common buzzards at a recovery center, the percentage of *E*. *faecium* isolates was also higher than that of *E*. *faecalis* (48.4% vs. 16.1%) [42]. In our cloacal samples, *E. casseliflavus* was found only in one case, but other authors found it in a high percentage of positive samples (26.6%), but the birds sampled did not include raptors [40].

The distribution of the susceptibility results of *Enterococcus* isolates to different antimicrobials according to sample type is shown in Table 2.

Eight isolates from the choana and nine from the cloaca were found to be resistant to IMP, but four of the isolates from the choana were identified as *E*. *faecium*, a species intrinsically resistant to this antibiotic. Considering this, 8% of *Enterococcus* species isolated from the choana and 17.6% from the cloaca were resistant to IMP. In a study conducted on raptors in the U.S.A. [38], 2% of isolates resistant to IMP were found, but no data about Enterococci species are available. Carbapenem resistance in bacteria isolated from wild birds is a matter of public health concern due to their potential transmission to the community.

Ampicillin-non-susceptible isolates were rarely found. In addition to this, only one of the non-susceptible isolates was identified as *E. faecalis* and none of them as *E. faecium*. It has been described [43] that almost 90% of *E. faecium* species isolated from humans are resistant to AM; however, almost all *E. faecalis* species remain susceptible. The disk diffusion method is considered a feasible manual technique for testing susceptibility to β-lactam antibiotics in Enterococci [44]. Our results agree with the ones found in other studies [38,39,40,41].

*E*. *faecalis* shows intrinsic resistance to SYN, and taking this into account, the results for resistance were 26.5% and 12.9% from the choana and cloaca, respectively. In common buzzards from recovery centers, a higher percentage of resistant *E. faecium* has been described [42]. Kwit et al. [40] found that 60% of *E. faecium* species isolated from wild birds were resistant to SYN.

As described in Table 2 displaying the results of Enterococci isolates, high percentages of isolates resistant to quinolones, especially to ciprofloxacin, were found in both choanal and cloacal samples. These results were higher than those previously described for Gram-negative bacteria in this population [21,22]. Significantly lower percentages of Enterococci isolates resistant to ciprofloxacin have been described by different authors [39,40]. This fact can be explained because in their studies, birds with different feeding habits were included, and most of them were not feeding in carrion. Common buzzards are also birds of prey, and in fecal samples of this species, about 30% of Enterococci isolates were found to be resistant to ciprofloxacin, but the animals had a diet of rabbits, small mammals, snakes, and lizards [42].

The percentages of non-susceptibility to VAN using the disk diffusion technique were similar in choanal and cloacal isolates (36% and 39.2%). These results were not confirmed when tested using E-test**^®^**: only one isolate was considered non-susceptible, accounting for less than 1% of the isolates (*E*. *faecium* from the cloaca, MIC = 8 µg/mL). Marrow et al. [38] found that 12% of Enterococci showed phenotypically intermediate susceptibility to vancomycin in raptors. An analysis of fecal samples from dead raptors from recovery centers showed a high percentage of VAN-resistant isolates (11.76%) [41]. They also found a high percentage of teicoplanin-non-susceptible isolates (48.83%) but including all types of wild birds’ samples analyzed. When common buzzards’ fecal samples from recovery centers in Portugal were studied, no glycopeptide-resistant Enterococci were found [42]. No glycopeptide-resistant Enterococci were found in samples from non-raptor wild birds [40].

Resistance to linezolid is also remarkable. Even when non-susceptible isolates account for only 8% of all isolates, this antibiotic is the option for the treatment of vancomycin-resistant Enterococci and methicillin-resistant *S. aureus* [32,45]; therefore, increasing non-susceptibility should be carefully monitored. Surprisingly, in a study including synanthropic, aquatic, and wild birds (about 60% from recovery centers), 51.25% of Enterococci isolates showed non-susceptibility to linezolid, a result that is a matter of concern [41]. In contrast, ref. [40] did not find any *Enterococcus* isolates resistant to LZD.

High-level resistance to gentamicin (HLRG) and high-level resistance to streptomycin HLRS were tested, showing no isolates with HLRG and only 5.9% with HLRS. Important differences were found in the study of Cagnoli et al. [41], who described a 23.53% rate of HLGR and a 58.82% rate of HLRS among Enterococci isolates, probably due to these animals being from animal recovery centers.

MDR profiles were found in 36 isolates (35.6%), including 15 from the choana and 21 from the cloaca. Among them, 24 were *E. faecalis* (32.9%), 8 were *E. faecium* (47%), and 5 were *E. gallinarum* (55.5%). Twenty-eight different profiles were observed, suggesting a diverse origin for these isolates. Canarian Egyptian vultures feed mostly in SFSs. Seven isolates were susceptible to all antimicrobials tested.

No studies have explored the presence of residues of veterinary drugs or MDR bacteria in carcasses used to feed Canarian Egyptian vultures.

The percentage of MDR isolates is clearly lower than the one described for Enterococci isolated from raptors’ fecal samples obtained from dead animals at recovery centers (94.12%) [41]. Among 92 Enterococci isolates obtained from different wild birds [40], only 1 was considered MDR. The role of wildlife in the spread of antibiotic resistance has been described in the literature in migratory and non-migratory animals [46,47]. This transmission poses a “One Health” challenge by contributing to the spread of antibiotic-resistant bacteria [48]. Avian scavengers feeding at landfills have been described as “maintenance hosts” of MDR bacteria and AMR genes of critical importance for human health [49,50], showing the relevance of a “One Health” perspective when addressing the antimicrobial resistance problem.

Our work demonstrates the presence of antibiotic-resistant enterococci, including multidrug-resistant strains, in an endangered subspecies of vultures. This may pose not only a risk for the spread of bacteria and resistance genes but also a problem for the treatment of infections in this subspecies. In addition, there is a possibility that these microorganisms could be transmitted to other protected animals in wildlife recovery centers. In the future, this study could be complemented by the detection of AMR genes, either by qPCR or by culture and WGS [51], in selected samples, including samples from other animals, as well as from human and environmental sources.

## 4. Conclusions

Further studies would be necessary to elucidate the pathways of acquisition of antimicrobial-resistant Enterococci in chicks in nests. This could help to design measures to avoid the spread of MDR bacteria between humans, animals, and the environment. From a conservation point of view, we have to be aware of the possibility of these bacteria causing difficult-to-treat infections in endangered wild bird species, as it has been described in poultry.

## Figures and Tables

**Table 1:** Antimicrobial categories and agents used to define MDR *Enterococcus* spp. (modified from Magiorakos et al. [4]).

Antimicrobial Categories	Antimicrobial Agents	Abbreviation and the Charge of Disks or MIC Breakpoint
Aminoglycosides except Streptomycin	Gentamicin (high level)	GM (500 µg)
Streptomycin	Streptomycin (high level)	S (2000 µg)
Carbapenems	Imipenem	IMP (10 µg)
Glycopeptides	Vancomycin Teicoplanin	VAN (30 µg) TEI (30 µg)
Oxazolidinones	Linezolid	LZD (30 µg)
Fluoroquinolones	Ciprofloxacin Levofloxacin	CIP (5 µg) LVX (5 µg)
Tetracyclines	Doxycycline	D (30 µg)
Penicillins	Ampicillin	AM (10 µg)
Streptogramins	Quinupristin/Dalfopristin	SYN (15 µg)

**Table 2:** Distribution of antibiotic resistance results for all isolated Enterococci according to sample type (choana/cloaca).

Sample Type/Antibiotic	Choana (n = 50) R/I/S (% R + I)	Cloaca (n = 51) R/I/S (% R + I)	Isolates with Resistance in Both Samples from the Same Animal
Imipenem	8/0/42 (16.0%)	9/0/42 (17.6%)	1
Ciprofloxacin	17/27/6 (88.0%)	21/23/7 (86.3%)	7
Levofloxacin	6/19/25 (50.0%)	13/14/24 (52.9%)	2
Teicoplanin	0/7/43 (14.0%)	2/8/41 (19.6%)	0
Linezolid	0/2/48 (4.0%)	1/5/45 (11.8%)	0
Ampicillin	1/0/49 (2.0%)	5/0/46 (9.8%)	0
Quinupristin/Dalfopristin *	28/4/6 (84.2%)	27/2/2 (93.5%)	15
Doxycycline	11/3/36 (28.0%)	10/12/29 (43.1%)	3
Vancomycin	5/13/32 (36.0%)	10/10/31 (39.2%)	1
Gentamicin	0/0/50 (0.0%)	0/0/51 (0.0%)	0
Streptomycin	5/0/45 (10.0%)	1/0/50 (2.0%)	0

* Since the manufacture of disks of this antibiotic has been discontinued, it was not possible to study all the isolates. R = resistant; I = intermediate; S = susceptible.

## Data Availability

Data can be provided upon reasonable request from the corresponding author.
